# A Depth Camera–Based, Task-Specific Virtual Reality Rehabilitation Game for Patients With Stroke: Pilot Usability Study

**DOI:** 10.2196/20916

**Published:** 2021-03-24

**Authors:** Yangfan Xu, Meiqinzi Tong, Wai-Kit Ming, Yangyang Lin, Wangxiang Mai, Weixin Huang, Zhuoming Chen

**Affiliations:** 1 The First Affiliated Hospital of Jinan University Guangzhou China; 2 The Sixth Affiliated Hospital of Sun Yat-sen University Guangzhou China; 3 School of Medicine Jinan University Guangzhou China; 4 Guangzhou Sanhao Computer Technology Co Ltd Guangzhou China

**Keywords:** virtual reality, rehabilitation, stroke, lower extremity, rehabilitation game

## Abstract

**Background:**

The use of virtual reality is popular in clinical rehabilitation, but the effects of using commercial virtual reality games in patients with stroke have been mixed.

**Objective:**

We developed a depth camera–based, task-specific virtual reality game, Stomp Joy, for poststroke rehabilitation of the lower extremities. This study aims to assess its feasibility and clinical efficacy.

**Methods:**

We carried out a feasibility test for Stomp Joy within representative user groups. Then, a clinical efficacy experiment was performed with a randomized controlled trial, in which 22 patients with stroke received 10 sessions (2 weeks) of conventional physical therapy only (control group) or conventional physical therapy plus 30 minutes of the Stomp Joy intervention (experimental group) in the clinic. The Fugl-Meyer Assessment for Lower Extremity (FMA-LE), Modified Barthel Index (MBI), Berg Balance Scale (BBS) score, single-leg stance (SLS) time, dropout rate, and adverse effects were recorded.

**Results:**

This feasibility test showed that Stomp Joy improved interest, pressure, perceived competence, value, and effort using the Intrinsic Motivation Inventory. The clinical efficacy trial showed a significant time-group interaction effect for the FMA-LE (*P=*.006), MBI (*P*=.001), BBS (*P*=.004), and SLS time (*P*=.001). A significant time effect was found for the FMA-LE (*P*=.001), MBI (*P*<.001), BBS (*P*<.001), and SLS time (*P*=.03). These indicated an improvement in lower extremity motor ability, basic activities of daily living, balance ability, and single-leg stance time in both groups after 2 weeks of the intervention. However, no significant group effects were found for the FMA-LE (*P*=.06), MBI (*P*=.76), and BBS (*P*=.38), while a significant group interaction was detected for SLS time (*P*<.001). These results indicated that the experimental group significantly improved more in SLS time than did the control group. During the study, 2 dropouts, including 1 participant who fell, were reported.

**Conclusions:**

Stomp Joy is an effective depth camera–based virtual reality game for replacing part of conventional physiotherapy, achieving equally effective improvement in lower extremity function among stroke survivors. High-powered randomized controlled studies are now needed before recommending the routine use of Stomp Joy in order to confirm these findings by recruiting a large sample size.

## Introduction

There are 800,000 new or recurring incidences of stroke annually in the United States; the number is rising as the population ages. More than half of stroke survivors live with at least one type of motor impairment [1]. In China, there are approximately 2 million incidences of a stroke every year. Among these stroke survivors, 70% to 80% cannot live independently as a result of multiple impairments, such as motor impairments with loss of strength, stereotypic movements, changes in muscle tone, and limitations in activities [2]. For many patients with stroke, balance and weight shift management constitute a risk for secondary injury. Lower extremity (LE) functional deficits in patients after stroke have aroused a great amount of attention because they play a vital role in stroke survivors’ quality of life [1,2]. Although stroke (new and recurring) remains prevalent, the number of available therapists is far from meeting the need, since the development of physical therapy has still not matured [3,4]. Rehabilitation technologies have the potential to increase the intensity and dose of rehabilitation, improve access to rehabilitation, reduce the workload of therapists, measure and provide feedback about performance and recovery, and engage and motivate patients [5-7]. Evidence-based medicine shows that high-intensity, repetitive, task-specific training tends to benefit patients greatly [8]. However, it is difficult to implement high-intensity, repetitive, task-specific training in a real clinical setting for a variety of reasons, including limited necessary resources and difficulty maintaining patients’ interest. Therefore, virtual reality–based gaming systems have become popular in medical rehabilitation and can be used as a novel alternative therapy method for motor recovery after stroke.

Kinect (Microsoft) is the leader in commercially available low-cost virtual reality (VR) hardware. This is because most of the Kinect’s games are aimed at the average person, and there are many more games designed by research teams for people with stroke, especially for upper limb motor function. However, there are few games focused on lower limb motor function [9]. VR, also known as immersive multimedia or computer-simulated reality, is a computer technology that replicates an environment, real or imagined, and simulates the user’s physical presence and environment to allow for user interaction and immersion. Virtual realities artificially create sensory experience, which can include sight, touch, hearing, and smell [10]. VR systems consist of a development platform, display system, interaction system, and integrated control system [11]. To realize the complete information interaction between computers and humans, normally we need some external device or devices to record the user’s movements. Among the kinds of external devices are force or tactile feedback systems, position trackers, data gloves or 6-degrees-of-freedom space mice, joysticks, and the Kinect sensor [11-13]. Kinect allows users to play without holding a game controller, which means they will not be bothered by wearing sensors that can be intrusive. This also saves time. Zhu et al [14] showed that the Kinect motion capture system was reliable and that the correlation coefficient of the dynamic track was quite good. A large number of clinical studies have shown that the accuracy of the Kinect somatosensory technology sensor for posture control and evaluation can fully meet the needs of body motion evaluation [15-17]. Eltoukhy et al [18] indicated that Kinect-based assessment might provide clinicians a simple tool to simultaneously assess reach distances while developing a clearer understanding of lower extremity movement patterns. Park et al [19] showed that the use of additional VR training with the Xbox Kinect gaming system was an effective therapeutic approach for improving motor function during stroke rehabilitation.

However, these systems were not specifically developed for patients after stroke, and those training sessions might produce multiple effects [20]. Those studies did not assess the flow experience of users, and few of them conducted a clinical randomized controlled trial. To address these issues, we developed a depth camera–based game, Stomp Joy, specifically for the lower limbs of patients with stroke. We also applied two principles of game design that are highly relevant to rehabilitation. The aim of this study was twofold: (1) develop a depth camera–based, task-oriented rehabilitation game for patients with stroke and (2) assess its usability and conduct a pilot study for stroke survivors’ LE rehabilitation.

## Methods

### Depth Camera–Based, Task-Oriented Rehabilitation Game

**Figure 1:**
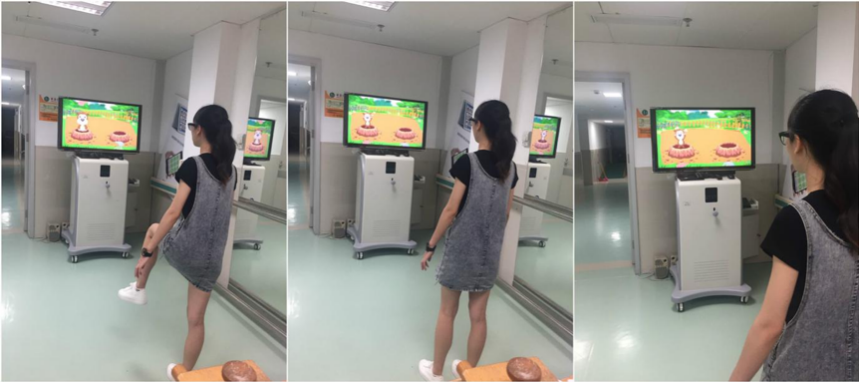
A participant experiencing Stomp Joy.

We developed a depth camera–based, task-specific rehabilitation game called Stomp Joy, which provides an enjoyable game for people with stroke in a rich interactive rehabilitation setting. The system is shown in Figure 1. The patient stands in front of a monitor facing an OpenNI–compliant depth sensor, the PrimeSense 3D Awareness Sensor (Apple Inc) with infrared projectors combined with standard RGB and infrared complementary metal-oxide semiconductor (CMOS) image sensors. The sensor has an effective angle of 70, a distance range of 0.8 to 3.5 m from where it is located, and a response time of 10 ms. A computer operated by Windows 7 with a 3.1-GHz quad-core central processing unit and 4 GB of SDRAM renders the images onto a 46-inch monitor with a resolution of 1920 × 1080 pixels. Its normal operating conditions are an environment temperature of 10 C to 35 C and a relative humidity of 35% to 75%. Stomp Joy can be operated by both the physical therapist and the patient via a local area network, providing control of the patients’ training modules and the level of difficulty.

Prior to each intervention session, a physiatrist responsible for the patient’s clinical situation outlined the customized training and gaming tasks, which were then further modified by the physical therapists during the actual training sessions.

The main user interface for Stomp Joy comprises 3 elements: (1) an introduction module that contains instructions about how the participant should play this game; (2) a rehabilitation curricular design module that contains different choices of difficulties for therapists and patients to choose; and (3) a rehabilitation game, Stomp Joy, that provides a repeated exercise motion using gaming concepts.

**Figure 2:**
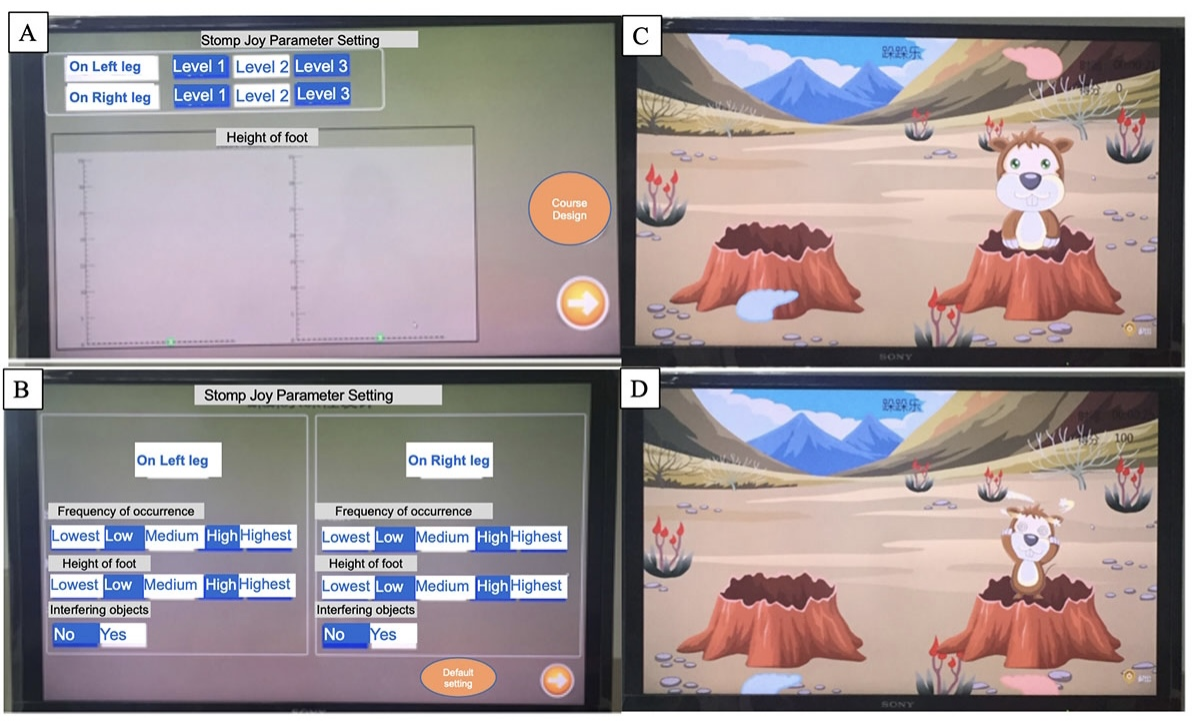
(A) The 3 difficulty levels for the left and right feet and the unique lifting foot height recognition interface. (B) The system-specific adjustment of the curriculum difficulty’s design according to patient conditions and default settings. (C) The patient raises their right foot, and when the hip and knee flexion reach the requirements of this level, the footprints above the gopher will turn from white to red. (D) When patients step down with the right foot, it shows an image of the gopher fainting.

Stomp Joy was created to meet patients’ need to improve their ability to transfer weight and recover to normal gait. In this game, the patient needs to lift their legs alternately to achieve the goal of transferring their center of gravity while supporting themselves on a single leg, with hip, knee, and ankle in coordination during the initial, middle, and end stages of the walk cycle. Stomp Joy was designed to increase lower limb control, endurance, speed, accuracy, range of motion, and trunk movements from synergistic motion patterns. The patient is asked to stand in front of the monitor and lift a foot to step on gophers by performing hip flexion and extension, knee flexion and extension, and single-leg support. Here, the frequency of gophers appearing on the display, the height of the gophers, and whether interference objects appear are controlled by therapists and patients, which means both therapists and patients (instructed by therapists) could select different difficulty levels of the game according to the patient’s performance. Game control and operation is very intuitive. The patient performs the lower limb exercise by mimicking stepping on gophers. The position of the footprint is reflected by the flexion of the hip joint; the bigger the flexion angle, the higher the footprint. The falling of the footprint was controlled by changing the flexion angle of the knee joint. When the color of the footprint changes to pink from white, which means that the hip and knee joint flexion angles both meet the requirement, it triggers a stampede action, and the patient can then complete a successful stomp. The game has gentle and pleasant background music. When the successful completion of a stomp happens, a gong sound plays. When the patient cannot complete the stomping successfully, the gopher on the screen plays a mocking sound for feedback (Figure 2).

### Participatory Design and Usability Test

Patients with stroke, physical therapists, and physiatrists were involved in the design of Stomp Joy. During the development process, we categorized and applied the feedback and suggestions from 3 representative user groups. Then, we carried out a usability test to assess Stomp Joy from the view of each stakeholder group. The patients with stroke performed 30-minute Stomp Joy sessions at regular intervals 5 times a week for 2 weeks under the supervision of physical therapists and physiatrists.

Since the most important benefit of Stomp Joy is the engagement of patients, we assessed the ability of Stomp Joy to provide strong motivation and enjoyment, followed by optimal flow experience [21]. The Intrinsic Motivation Inventory (IMI) is a multidimensional measurement scale that can assess participants’ subjective experience, then apply a new activity in a laboratory environment [22-26]. To assess the experience provided by Stomp Joy, we examined 5 constructs shown by usability professionals to characterize the optimal flow state for new activities: interest and enjoyment, pressure and tension, perceived competence, value and usefulness, and effort and importance [23,27,28]. We excluded perceived choice and relatedness. To test if Stomp Joy afforded the patients with stroke a desirable level of rehabilitation, we conducted a usability test in 11 patients with stroke and collected their responses to whether they were highly engaged and considered the user experience pleasant so that they were further motivated to take an active part in the Stomp Joy intervention. We conducted a semistructured, one-on-one interview with the patients with stroke, physical therapists, and physiatrists. Based on the existing literature, 3 frameworks from implementation science (normalization process theory, conceptual framework for implementation fidelity, and the Consolidated Framework for Implementation Research) were used to address the study objectives [29]. The interviews were conducted by the first and second author. Interviews took place face to face at the worksite at a time suitable to the participants.

### Clinical Experiments

Patients with hemiparetic lower limb dysfunction secondary to first-ever stroke were recruited from the First Affiliated Hospital of Jinan University from January 2015 to September 2016. All patients exhibited mild to severe deficits of the paretic lower extremities (≥3 on the Brunnstrom stages of motor recovery for the proximal part of the lower extremity [30], the hip and knee flexion reach [31]). The exclusion criteria were preexisting lower limb impairment, any painful condition affecting the lower limbs, difficulty standing for at least 30 minutes, severe cognitive impairment (Mini-Mental State Examination score less than 22 points [32]), and severe aphasia. The exclusion criteria were kept to a minimum in order to evaluate the feasibility of the use of Stomp Joy among a variety of patients. All patients provided written informed consent to participate and written informed consent for the publication of their clinical image. The study was conducted in accordance with the Declaration of Helsinki and approved by the Institutional Review Board of Jinan University. The individual in Figure 1 also gave permission to publish their image.

**Figure 3:**
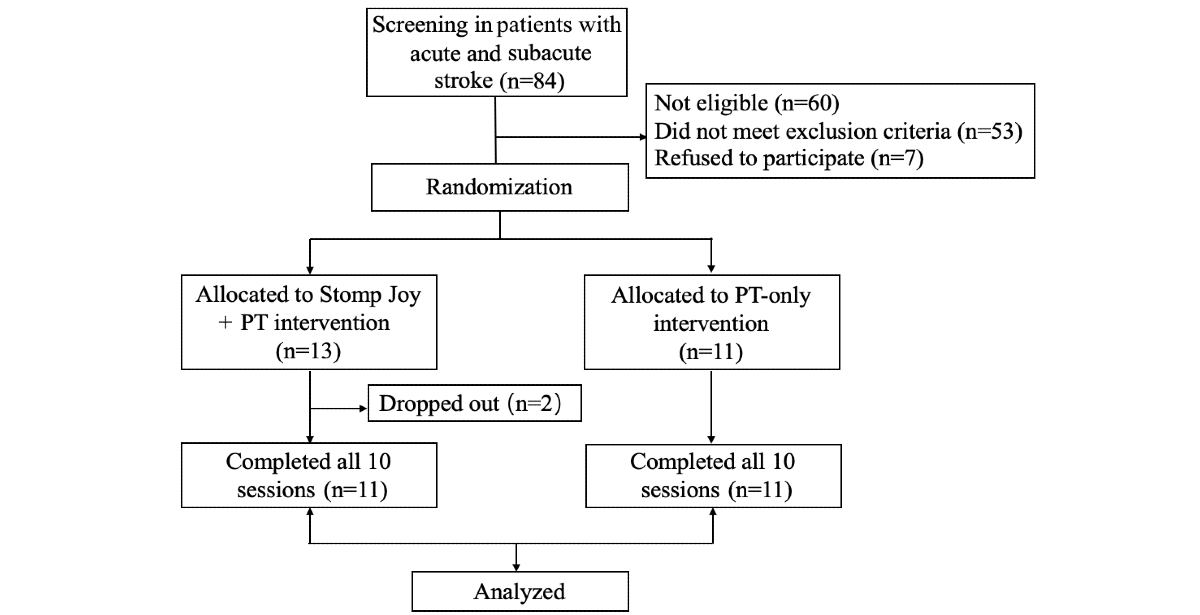
Flowchart of the study. PT: physical therapy.

A prospective single-blind pilot study was conducted in patients with acute and subacute stroke. We randomized the patients to receive 10 sessions over 2 weeks of either conventional physical therapy (PT) plus 30 minutes of Stomp Joy training (Stomp Joy + PT group) or conventional physical therapy alone (PT-only group). The PT was delivered for 30 minutes by a trained physical therapist who was blinded to the protocol in order to provide participants the same PT content in the conventional clinical setting. The trained assessor was also blinded to patients’ allocation and randomization to minimize assessor bias. The primary outcome was the Fugl-Meyer Assessment for Lower Extremity (FMA-LE) [33], and the secondary outcomes were the Modified Bathel Index (MBI) [34], Berg Balance Scale (BBS) [35], and single-leg stance (SLS) time [36]. The SLS time was collected 3 times by recording the endurance time of standing on the paretic limb. The patient could not touch any object while supporting themselves on one leg, and the average time was calculated after 3 repetitions were measured. Between each repetition, the patient got 1 minute to rest. These assessments were made at baseline and during the last session (tenth session) by evaluators who were blinded to the type of intervention. Adverse effects related to the Stomp Joy intervention and the number of patients who dropped out during the study period were also recorded (Figure 3).

### Statistical Analysis

One-sample 2-tailed *t* tests against the neutral value in the 7-point Likert rating were used to assess the responses to the 5 domains of the IMI [22]. A mean rating above 4 indicated that on average the patients agreed rather than disagreed with the statement.

Data were analyzed using IBM SPSS version 24.0 (IBM Corp). The baseline data (height, weight, age, time since stroke, type of stroke [hemorrhage or infarction], region of stroke [left or right]) between the two groups were compared using 2 independent-sample *t* tests and Fisher exact tests (gender). The main effects of time, group, and time-group interaction were analyzed using repeated-measures one-way analysis of variance (ANOVA) with a Greenhouse-Geisser correction to compare the changes in the FMA-LE, BBS, and MBI scores and the SLS time between the experimental group and control group. The level of statistical significance was *P*<.05 for all comparisons.

## Results

### Participatory Design and Usability Test

We interviewed 3 representative user groups (ie, patients with stroke, physical therapists, and physiatrists) to collect the key elements of an interactive VR rehabilitation system (Table 1). We prioritized and incorporated those key elements into Stomp Joy.

**Table 1:** Key elements of a depth camera–based, task-specific virtual reality rehabilitation game [37].

Component	Key elements
Device	OpenNI–compliant depth sensor, stable system
Design	Goal-oriented task-specific contents; interactive and interesting elements; and easy-to-understand tutorials to show explanation, evaluation, and training and increase motivation for rehabilitation
Difficulty level	Easy to know how to interact, adjustable to match individualized performance
Scoring	Scoring system to record and compare performance status
Sound	Sound showing feedback of the performance, exaggerated effects sounds to improve motivation

The advantages reported by each user group after initial testing of Stomp Joy included that it provided a new immersive experience for patients with stroke and improved their motivation and attention, released part of the burden on physical therapists and helped them manage the intervention programs, and helped physiatrists conduct an effective individualized intervention.

Table 2 shows the scores across the 5 main components of the flow experience (ie, interest and enjoyment, pressure and tension, perceived competence, value and usefulness, and effort and importance), which exhibited a consistent pattern. For all 5 subscales, the patients with stroke gave higher ratings. They found that the Stomp Joy training was enjoyable and useful. Additionally, they felt they exerted effort when playing the game.

**Table 2:** IMI of the Stomp Joy intervention among patients with stroke.^a^

IMI^b^ subscale	Rating, mean (SD)	t test (*df*)	*P* value
Interest and enjoyment	5.91 (1.09)	5.8 (10)	<.001
Pressure and tension	1.77 (0.90)	–8.17 (10)	<.001
Perceive competence	5.50 (1.00)	4.98 (10)	.001
Value and usefulness	5.77 (1.03)	5.69 (10)	<.001
Effort and importance	5.32 (1.27)	3.44 (10)	.006

^a^All of the statements used in this study were deliberately rephrased in positive terms that the patients could easily understand.

^b^IMI: Intrinsic Motivation Inventory [22].

To see if the physical therapists could easily apply Stomp Joy, we collected suggestions from 5 physical therapists who took part in Stomp Joy through semistructured interviews. Of the 5 physical therapists, 4 strongly agreed with the following statements: “I can easily allocate the rehabilitation program according to the evaluation via Stomp Joy to patients with stroke,” “I can easily manage the prescription using Stomp Joy,” and “I can tell from patients’ faces that they were glad to see that their feet on the screen could do more than in reality.” One of the physical therapists reported, “The game was a little bit repetitive, so after 10 sessions, patients could not maintain their interest.” This physical therapist also reported that the system did not have a recording system that they could track each patient’s performance.

To evaluate whether Stomp Joy played a meaningful rehabilitation role for patients with stroke, we conducted semistructured interviews with 5 physiatrists who took part in the Stomp Joy intervention. Of the 5 physiatrists, 3 reported that Stomp Joy seemed to be an effective and easy way to administer PT and that it seemed to be more efficient when conducted in the clinic. Still, 1 physiatrist reported that Stomp Joy needs to be supervised by a therapist and thus would not help save therapy resources. Another physiatrist reported that Stomp Joy needs to expand its game content so that it could be suitable for more situations.

### Clinical Experiment

In all, 84 patients with stroke were screened for the study, 53 patients with stroke were excluded because they met exclusion criteria, and 7 declined to participate. Hence, 24 patients with stroke were recruited. They were randomly allocated into the Stomp Joy + PT intervention group or the PT-only intervention group by picking a sealed envelope. Among the participants in the Stomp Joy + PT intervention group, 2 patients did not complete the full study. One of the patients discontinued because they fell down during training with Stomp Joy, although the patient was unharmed. One of the patients dropped out because of personal issues unrelated to any adverse effects of Stomp Joy. None of the patients who participated in the Stomp Joy intervention suffered from any adverse effects that would be likely to result from VR, such as dizziness or disorientation.

None of the baseline characteristics differed significantly between the two groups (Table 3). The Fisher exact test was performed for sex composition, type of stroke, and lesion side of the experimental group and control group, and 2 independent-sample *t* tests were performed for age, height, and weight. The results showed that there was no significant difference in height (*P*=.19), weight (*P*=.52), age (*P*=.53), or disease duration (*P*=.93) between the two groups. The Fisher exact probability method showed no significant difference in gender composition between the two groups (*P*=.39). The general statistics of the patients are shown in Table 3.

**Table 3:** Baseline characteristics of the patients with stroke (N=22).

Outcome	Control group (n=11)	Experimental group (n=11)	*P* value^a^
Male, n (%)	8 (73)	5 (46)	.39
Hemorrhage, n (%)	6 (55)	5 (46)	.76
Right-side lesion, n (%)	9 (82)	10 (91)	.61
Height (cm), mean (SD)	167.09 (4.46)	162.72 (5.97)	.19
Weight (kg), mean (SD)	67.27 (7.93)	60.54 (9.39)	.52
Age (years), mean (SD)	52.82 (12.29)	57.55 (14.22)	.53
Time since stroke (months), mean (SD)	4.91 (3.21)	3.81 (3.60)	.93
FMA-LE^b^, mean (SD)	18.36 (5.52)	22.64 (4.61)	.11
MBI^c^, mean (SD)	70.91 (16.10)	70.45 (17.39)	.51
BBS^d^, mean (SD)	35.82 (10.32)	37.73 (8.79)	.62
SLS^e^ time (s), mean (SD)	1.09 (0.61)	1.04 (0.61)	.86

^a^All *P* values were for paired *t* tests except for gender (male or female), for which *P* value was for Fisher test. Statistically significant at *P*<.05*.*

^b^FMA-LE: Fugl-Meyer Assessment for Lower Extremity.

^c^MBI: Modified Barthel Index.

^d^BBS: Berg Balance Scale.

^e^SLS: single-leg stance.

As determined by the Shapiro-Wilk test, the FMA-LE, MBI, BBS, and SLS time data of each group obeyed normal distribution (*P*>.05). Repeated-measures ANOVA with a Greenhouse-Geisser correction showed a significant effect of time on FMA-LE (*P*=.001; ηp^2^=0.69), MBI (*P*<.001; ηp^2^=0.82), BBS (*P*<.001; ηp^2^=0.93), and SLS time (*P*=.03; ηp^2^=0.41) (Table 4). These results indicate that the experimental group and control group improved LE physical function, MBI, BBS, and SLS time after 2 weeks of intervention. However, no significant group effects were found for FMA-LE, MBI, or BBS, while a significant group interaction was found for SLS time (*P*<.001; ηp^2^=0.82) (Table 4). These results indicate that the experimental group significantly improved more in SLS time than the control group. The time-group interaction also showed significant effects for FMA-LE (*P*=.006; ηp^2^=0.55), MBI (*P*=.001; ηp^2^=0.66), BBS (*P*=.004; ηp^2^=0.59), and SLS time (*P*=.001; ηp^2^=0.68) (Table 4). This suggests that the experimental group had better outcomes after the intervention than the control group.

**Table 4:** Main effect of time, group, and time-group interaction of the intervention on the outcome measures by repeated-measures analysis of variance with a Greenhouse-Geisser correction.

Parameters		Study group					*P* value^a^ (ηp^2^)				
		Control group, mean (SD)		Experimental group, mean (SD)		Time			Group		Interaction
**FMA-LE^b^**						.001 (0.69)			.06 (0.32)		.006 (0.55)
	Week 0	18.36 (5.52)	22.64 (4.61)								
	Week 2	19.82 (5.51)	25.73 (4.45)								
**MBI^c^**						<.001 (0.82)			.76 (0.01)		.001 (0.66)
	Week 0	70.91 (16.10)	70.45 (17.39)								
	Week 2	75.00 (15.17)	80.00 (16.59)								
**BBS^d^**						<.001 (0.93)			.38 (0.08)		.004 (0.59)
	Week 0	35.82 (10.32)	37.73 (8.79)								
	Week 2	38.00 (10.61)	43.45 (9.30)								
**SLS^e^ time**						.025 (0.41)			<.001 (0.82)		.001 (0.68)
	Week 0	1.09 (0.61)	1.04 (0.61)								
	Week 2	1.53 (0.71)	3.63 (1.79)								

^a^Statistically significant by repeated-measures analysis of variance.

^b^FMA-LE: Fugl-Meyer Assessment for Lower Extremity.

^c^MBI: Modified Barthel Index.

^d^BBS: Berg Balance Scale.

^e^SLS: single-leg stance.

## Discussion

### Implications and Future Studies

Because there is damage in the advanced central nervous system in patients with stroke, the normal synaptic connections are broken and the low central nervous system is not under control, resulting in the loss of muscle strength and coordination, body balance dysfunction, impaired lower limb movement function, and other symptoms [38]. Based on the depth camera somatosensory interactive technology, the virtual reality game can realize the natural interaction between the user and the virtual environment, which can combine spatial vision, perception movement, task concept, motion control, and feedback information to improve the posture control and balance function of patients with stroke [15,39,40].

Stomp Joy is a depth camera–based, task-specific virtual reality rehabilitation game developed to facilitate motor recovery after stroke. At the beginning of the game, patients need to observe the action, which contributes to motor recovery through mirror motor neuron activation [41], as viewing the movements demonstrated on the screen might also help with functional improvement. In addition, this somatosensory interactive virtual reality technology allows the patient to control the training independently by using their own gestures to control the interface and the game process. Thus, it might raise the user’s awareness of their own motion. Adapting the intervention to patients’ current functional levels and providing appropriate therapy programs can lead to patients’ functional improvement. The physical therapist can adjust the stimulus according to the patient’s functional level and the feedback in Stomp Joy to motivate patients to engage more. Training is considered meaningful when there is a good relationship between the patient’s movements and the outcome on the system.

Rehabilitation goals should also be considered. Stomp Joy is able to gradually adjust the difficulty level according to the patient’s progress, which is a valuable feature. On the other hand, the physical therapist, who is in direct contact with the patient, could combine the training performances and the practice date provided by Stomp Joy to design new sets of individualized tasks for patients. Hence, we made sure that patients with stroke could continue to be optimally challenged as the training continues. We also created a safe testing and training environment for patients based on the depth camera’s virtual reality interactive technology. Because of its unique training format, this intervention is more attractive to patients compared with traditional training, which in turn improves patient initiative in rehabilitation and encourages patients to actively participate in rehabilitation.

Another area of concern in the real-world setting is how to deliver the treatment safely. It is always important to ensure the patients’ safety when applying a new training system. While Stomp Joy performed adequately, 1 patient fell during treatment. Afterward, we placed a frame beside the patients, and no other patients fell during treatment with Stomp Joy. The motor function of patients who are recruited into the training should also be strictly monitored, and the physical therapist should supervise the patients during the entire treatment.

According to the semistructured interviews of physical therapists and physiatrists, it would be better to add a records module of patients’ training data in the Stomp Joy system. Additionally, more games should be designed to avoid monotony during long-term training. To improve the whole system and its application in the clinic, further studies are needed.

### Limitations

There are several limitations to our study. First, different experimental protocols using different intervention times in the two groups might have caused the inconsistency in the results. However, the goal of the experiment was not to prove that treatment with the Stomp Joy VR game is better than traditional physical therapy. The goal of the experiment was to introduce the newly designed depth camera–based VR game, Stomp Joy, and to see if the new style of treatment, together with traditional therapy, could bring any progress to the patients. In addition, there have been many studies that have applied VR games for different amounts of time [42]. In the near future, an investigation focused on the duration of time with a consistent protocol will be needed in order to establish an appropriate rehabilitation protocol. Second, although the accuracy of the depth sensor (PrimeSense 3D Awareness Sensor with infrared projectors combined with standard RGB and infrared CMOS image sensors) in the Kinect system was discussed in a previous study [14], in which the maximal static error was 2.76° and the correlation coefficient of the dynamic track was 0.9917 when tracking lower extremity movement, we did not study the accuracy of the tracking of lower extremity movement in this game system, for which a further study is needed.

In the clinical experiment, the assessments were restricted to functional outcomes (FMA-LE, MBI, and BBS) and objective measurements (SLS time). It was better to analyze the improvement separately according to different side impairments, but in this pilot study, the majority of patients had lesion sides on the right (Table 3). More participants need to be recruited to see whether unilateral impairment could influence the functional outcomes. This study would have been improved by using more objective measurements, such as range of motion and strength. It also would have been better to evaluate the kinematic data recorded in real time during the Stomp Joy intervention. In addition, factors such as cognitive function, flow experience, motivation, and depression were not appraised in the clinical experiment, which is common when examining patients with stroke.

### Conclusion

This study described a pilot clinical trial of a depth camera–based, task-specific virtual reality rehabilitation game called Stomp Joy. Stomp Joy was proven to be a feasible and safe rehabilitation tool to enhance lower limb motor function and balance among patients with stroke. Stomp Joy can also encourage the patient’s functional development, improve immersion, and motivate further rehabilitation by providing meaningful play, optimal challenge, and flow experience. However, because this was a pilot study that was underpowered to show superiority of one treatment over another and had several limitations, further powered randomized controlled trials are now needed before recommending routine use of Stomp Joy in order to confirm these findings by recruiting a larger sample size.

## Abbreviations

ANOVA: analysis of variance
BBS: Berg Balance Scale
CMOS: complementary metal-oxide semiconductor
FMA-LE: Fugl-Meyer Assessment for Lower Extremity
IMI: Intrinsic Motivation Inventory
LE: lower extremity
MBI: Modified Barthel Index
PT: physical therapy
SLS: single-leg stance
VR: virtual reality
